# Sepsis-induced acute kidney injury

**DOI:** 10.4103/0972-5229.63031

**Published:** 2010

**Authors:** Arghya Majumdar

**Affiliations:** **From:** Department of Nephrology, AMRI Hospitals, Kolkata, India

**Keywords:** Sepsis, acute kidney injury, AKI, acute renal failure, extracorporeal purification of blood

## Abstract

Acute kidney injury (AKI) is a common sequel of sepsis in the intensive care unit. It is being suggested that sepsis-induced AKI may have a distinct pathophysiology and identity. Availability of biomarkers now enable us to detect AKI as early as four hours after it's inception and may even help us to delineate sepsis-induced AKI. Protective strategies such as preferential use of vasopressin or prevention of intra-abdominal hypertension may help, in addition to the other global management strategies of sepsis. Pharmacologic interventions have had limited success, may be due to their delayed usage. Newer developments in extracorporeal blood purification techniques may proffer effects beyond simple replacement of renal function, such as metabolic functions of the kidney or modulation of the sepsis cascade.

## Introduction

Sepsis, a commonly encountered scenario in an intensive care unit (ICU), often leads to multi-organ dysfunction and the kidney is one of the organs frequently afflicted. Acute kidney injury (AKI) occurs in about 19% patients with moderate sepsis, 23% with severe sepsis and 51% with septic shock, when blood cultures are positive.[1]

The beginning and ending supportive therapy (BEST) kidney investigators highlighted the fact that sepsis is the most common cause of AKI in critically ill patients (47.5%), after evaluating a varied population, in 54 hospitals spread over 23 countries. They inferred that septic AKI was associated with greater derangement in hemodynamic and laboratory parameters, greater severity of illness and higher need for mechanical ventilation and vasopressor therapy. A few more facts emerged from this study. Oliguria was found to be more common in septic AKI (67 *vs.* 57%; *P* < 0.001). Septic AKI had a higher in-hospital mortality rate, compared with nonseptic AKI (70.2 *vs.* 51.8%; *p* < 0.001). Median duration of ICU and hospital stay for survivors (37 *vs.* 21d; *P* < 0.0001), was longer for septic AKI.[2]

Distinguishing between septic and non-septic AKI, therefore, may not just be of academic interest but may have clinical relevance for physicians. It has been suggested that septic AKI may have a distinct pathophysiology as well.[3] Thus, septic AKI may have a unique identity and responses to interventions and outcome may be different in this group of patients, when compared to those with non-septic AKI.

Significant progress has been made, over the years, towards learning how to detect AKI early, agreeing on an international consensus definition, delineating the pathophysiologic mechanisms which predispose to a high incidence of AKI in sepsis, trying to deduce logical protective and preventive strategies and finally on how to deliver the optimal renal support when the kidney fails.

This review will try to proffer a bird's eye view of the recent developments in this field and where we stand now.

## Diagnosis

Two classification systems – RIFLE (risk, injury, failure, loss, end-stage) criteria[4] and acute kidney injury network (AKIN) criteria[5] have been recently developed and widely validated, to diagnose and stratify patients with AKI. This may enable the development of clinically effective approaches to prevention and management and facilitate comparisons of their efficacy in different study populations.

## Early detection

Despite significant improvement in therapeutics, the mortality and morbidity associated with AKI remain high. A major reason for this is the lack of early markers for AKI, akin to troponins in acute myocardial disease, and hence an unacceptable delay in initiating therapy. Conventional urinary biomarkers such as casts and fractional excretion of sodium have been insensitive and nonspecific for the early recognition of AKI. Fortunately, the application of innovative technologies such as functional genomics and proteomics to human and animal models of AKI has uncovered several novel genes and gene products that are emerging as biomarkers. The most promising of these are a plasma panel [neutrophil gelatinase-associated lipocalin (NGAL)[6] and cystatin C[7]] and a urine panel [NGAL,[8] interleukin 18 (IL-18)[9] and kidney injury molecule 1 (KIM-1)[10]].

As they represent sequentially expressed biomarkers, it is likely that the AKI panels will be useful for timing the initial insult and assessing the duration of AKI. Based on the differential expression of the biomarkers, it is also likely that the AKI panels will distinguish between the various types and etiologies of AKI.

Moreover, new evidence indicates that the biomarkers may even be able to differentiate septic from non-septic AKI. In a study of 83 patients (43 with sepsis) in Melbourne, septic

AKI was associated with significantly higher plasma (293 *vs.* 166 μg/ml) and urine (204 *vs.* 39 μg/mg creatinine) NGAL compared with non-septic AKI (*P* < 0.001).[11]

## Pathophysiology

The exact pathophysiology of sepsis-induced AKI is not known, however, it is generally accepted that it has a multi-pronged injury pathway. This form of AKI has components of: ischemia-reperfusion injury, direct inflammatory injury, coagulation and endothelial cell dysfunction, and apoptosis.[12]

Moreover, based on recent evidence we may presume that the pathophysiologic mechanisms of sepsis-induced AKI are different from non-septic AKI.[13] This would translate to the issue that sepsis-induced AKI may entail different therapeutic strategies.

Gram-negative sepsis, which is more common in India, is independently associated with AKI.[14] An elevated plasma concentration of endotoxin (lipopolysaccharide; LPS) is often found in the systemic circulation during sepsis, regardless of the type of the infecting microorganism,[15] possibly as a result of the translocation of LPS originating from the resident Gram-negative flora of the gut.[16] During the inexorable downward spiral of sepsis, LPS, then cytokines, and consequently nitric oxide (NO) is released.

Adding to the translocation of intestinal-derived LPS that occurs during any form of sepsis, the multiplication and destruction of Gram-negative bacteria results in the release of LPS into the bloodstream and its rapid dissemination throughout the body.[17] LPS binds with the LPS-binding protein (LBP) through the biologically active component lipid A of LPS.[18] The LBP-LPS complex binds to the co-receptor CD14, which leads to interactions with the cell surface Toll-like receptor 4-MD-2 complex on monocytes, macrophages and neutrophils,[19] but this complex also binds to other cells, including renal tubular epithelial cells.[20] These cells are then stimulated to produce cytokines through a myeloid differentiation primary response gene (MyD88)-dependent and an MyD88-independent pathway.[21] Dear's group show that the initiation of septic AKI is dependant mainly on MyD88b.[22]

The proinflammatory cytokines induced upon LPS exposure, which include tumor necrosis factor (TNF)-, interleukin (IL)-1, and interferon (IFN)-, bind to their specific receptors on different cell types.[23] In the kidney, this takes the form of TNF receptor 1 on glomerular endothelial cells and TNF receptor 2 on renal tubular epithelial cells.[24] After a chain of reactions there is transcription of the inducible NO synthase (iNOS) gene, and the translation of iNOS mRNA, and the subsequent assembly of iNOS protein, which culminates in the formation of NO.[25]

The production of large amounts of NO during sepsis is responsible for systemic vasodilatation, which results in septic shock. The resultant arterial volume depletion is sensed by baroreceptors, which triggers increased sympathetic activity and angiotensin production. The end result is intrarenal vasoconstriction with sodium and water retention and a reduction in glomerular filtration rate (GFR).[26] In animal models it has been shown that LPS can cause renal failure in the absence of significant hypotension.[27]

On the other hand, recent animal models of hyper-dynamic sepsis (increased cardiac output along with a decreased blood pressure) reveal that sepsis-induced AKI can occur despite renal hyper- perfusion and intrarenal vasodilatation.[3] An intact renal blood flow does not assure adequate perfusion to microvascular beds, as shown by the reduction in cortical microvascular perfusion that occurs during systemic inflammation.[28,29]

Neither systemic nor intrarenal hemodynamic instability is the sole incriminating factor in sepsis-induced AKI. In fact, hemodynamic factors do not seem to be very significant, as hypotension does not correlate with AKI in critically ill patients with severe sepsis.[30] Further, the conjecture of direct toxic effects of LPS to renal proximal tubular cells *in vivo* is substantiated by findings from *in vitro* studies showing that lipid A of LPS is responsible for NO mediated oxidant injury.[31] The production of both cytokines and oxygen free radicals in systemic inflammation might also contribute to renal tubular injury.[26] Thus one may conclude that although the pathogenesis of sepsis-induced AKI is multi- factorial and has not completely been delineated, NO is felt to be a key player in this process.[32,33]

## Protective strategies

It is difficult to implement timely preventive strategies in sepsis as renal damage may have already occurred before signs of sepsis become overt. Though AKI has been seen to occur in the absence of hemodynamic compromise, it would seem rational to try and avoid any form of nephrotoxic insult, maintain effective intravascular volume and renal perfusion.

### Early goal-directed therapy

The surviving sepsis campaign recommends that extracellular volume and cardiac output be assessed and supported with adequate and early goal-directed therapy.[34] This includes volume and vasopressor support to achieve a mean arterial pressure 65 mm Hg and a central venous pressure of 8 to 12 mm Hg (or 12 to 15 mm Hg in patients who receive positive pressure ventilation). The importance of early goal-directed therapy was brought to the fore by Rivers *et al.*[35] who demonstrated that early *versus* delayed administration of fluid, vasopressors, blood products, and inotropes to maintain central venous oxygen saturation of >70% had important benefits in terms of mortality and multiorgan failure including AKI. These observations highlight the importance of early initiation of resuscitation. Fluid administration is essential to restore effective circulating volume but should stop when patients are no longer fluid responsive.

### Choice of resuscitation fluid

Choice along with timing and amount of fluid administration are also emerging as important determinants of AKI, with some concerns raised over the use of certain forms of colloid, namely hydroxyethyl starch. In a randomized study of septic patients, Schortgen *et al*.[36] found that patients who were randomly assigned to hydroxyethyl starch had a much higher risk of acute renal failure, oliguria, and higher peak creatinine than those who were randomly assigned to gelatin.

As far as albumin is concerned, results of the saline *versus* albumin fluid evaluation (SAFE) study, a randomized comparison of human albumin with crystalloid in the ICU, seem to indicate that albumin is safe, albeit no more effective than saline, for fluid resuscitation.[37] That being said, in a predefined subgroup with sepsis (approximately 18% of the total population), the SAFE study found a trend toward improved survival in the albumin group, with a relative risk of 0.87 (95% confidence interval 0.74 to 1.02; *P* = 0.09). This requires further study.

### Choice of vasopressor

Maintaining renal perfusion pressure is important. To achieve adequate renal perfusion pressure, fluid resuscitation is not enough and patients with sepsis often require vasopressor support. Norepinephrine seems to be the drug of choice when volume and cardiac output have been corrected and significant vasodilation impedes the achievement of an adequate renal perfusion pressure. Contrary to the concerns about vasoconstriction from norepinephrine leading to decreased renal perfusion and worsening renal function, the opposite has been demonsttrated[38] and norepinephrine is considered to be a first-line agent for the management of hypotension in sepsis. In septic shock, vasodilation, particularly through increased synthesis of nitric oxide, occurs through multiple mechanisms and may be hypo-responsive to catecholamines. Further, the presence of high levels of endogenous (and exogenous) catecholamines can lead to down-regulation of adrenoreceptors. Along with this the inappropriately low levels of endogenous vasopressin, have led to the notion of using exogenous vasopressin and its analogues in the management of septic shock. In a small, pilot, randomized, controlled trial of 24 patients with severe septic shock, the use of vasopressin led to improved urine output, an increase in creatinine clearance of approximately 75%, and decreased overall pressor requirement, whereas no such improvement was seen in the comparator arm of norepinephrine.[39] The vasopressin and septic shock trial (VASST) trial, though indicating a lesser mortality in the patients with less severe sepsis, did not show any difference in the incidence of AKI or need for renal replacement therapy with the use of vasopressin.[40]

### Intra- abdominal pressure

In the ICU, particularly after surgery or fluid resuscitation of septic patients, there is a chance of abdominal pressure increasing. This can cause abdominal compartment syndrome with pressure effects on the inferior venacava and resultant fall in renal perfusion pressure.[41] One must take preventive measures in this regard.

### Tight glucose control

The use of aggressive insulin therapy aimed at achieving normoglycemia in critically ill patients has been shown by the van den Berghe group to reduce mortality significantly in critically ill, surgical patients with sepsis.[42] Among the other important findings of this trial was a dramatic reduction in the development of severe AKI that required RRT (8.2 *versus* 4.8%; *P* = 0.04) and a reduction in the number of patients who experienced a peak creatinine >2.5 mg / dl or a peak urea nitrogen of >54 mg / dl. In a subsequent study from this group of medical patients in the ICU, mortality did not improve, but there was an important reduction in the risk for AKI defined by I or F criteria of RIFLE (8.9 *versus* 5.9%; *P* = 0.04).[43] A possible explanation for this finding may relate to the fact that insulin may play an important anti-inflammatory and anti- apoptotic role in sepsis. However, a very large, multicenter, randomized, controlled study to assess the effectiveness of intensive insulin therapy in critically ill patients, the NICE-SUGAR study,[44] showed no decrease in requirement for or number of days on renal replacement therapy with intensive glucose control and in fact showed a higher incidence of hypoglycemia and mortality in this group. The debate, therefore, continues.

### Low tidal volume ventilation

The acute respiratory distress syndrome (ARDS) network had shown us that low tidal volume ventilation for ARDS patients reduced mortality.[45] In a rabbit model of ARDS, Imai *et al.* went a step further to demonstrate that low tidal volume ventilation led to less apoptosis of tubular cells and resultant AKI.[46] The protective effect on the kidneys was attributed to Fas ligand.

## Pharmacologic interventions

Drugs which were felt to be protective for the kidneys like diuretics and ‘renal dose’ dopamine have fallen out of favor over time.

### Fenoldopam

Fenoldopam as a dopamine-1 receptor agonist has the potential to increase renal blood flow. In a prospective double blind placebo control trial of 300 patients with severe sepsis it was shown that prophylactic fenoldopam infusion reduced the incidence of AKI significantly.[47] The results of this study are promising but need to be reproduced in other centers.

### Activated Protein C

Activated protein C (APC) not only has a hindering effect on thrombin generation (anti- thrombotic) but is also an agonist of protease activated receptor-1 (PAR-1). This dual mechanism of action helps to modulate endothelial dysfunction by blocking cytokine signaling, adhesion molecule expression, vascular permeability, apoptosis and leucocyte migration.[48]

A retrospective analysis of the PROWESS trial[49] revealed that therapy with activated drotrecogin alfa was associated with improved renal function compared to placebo in patients who had severe protein C deficiency. Treatment with activated protein C (APC) reduced progression to renal failure as well as the need for renal replacement therapy.

### In the pipeline

Agents like N acetyl cysteine and atrial natriuretic peptide have been seen to be of some benefit in other models of AKI but their role in sepsis-induced AKI needs to be investigated. On the other hand, there is no consensus regarding the role and appropriate utilization of corticosteroids in septic shock. RCTs are needed to determine if it has a preventive role in septic AKI.

Promising agents that are in the development phase include: selective iNOS inhibition, toll-like receptor inhibition, IL-10 augmentation, modulators of the protein C pathway, caspase inhibitors, lysophosphatidic acid and mesenchymal stem cell mediated therapeutics.[50]

## Extracorporeal purification of blood

Blood can be purified by running it in an extracorporeal circuit through a device (membrane, sorbent) where solute (uremic toxins, cytokines) and fluid can be removed. In patients with sepsis it may help in two ways: renal replacement therapy and removal of inflammatory mediators, to achieve immune homeostasis.

### Indications

Indications for commencing renal replacement therapy (RRT) in sepsis-induced AKI are by and large similar to other forms of AKI. They are: worsening azotemia, refractory volume overload, severe metabolic acidosis, uremic encephalopathy and severe electrolyte disarray.[51] In patients with sepsis, sustained oliguria or severe metabolic acidosis may be reason enough to start RRT as these patients often do not manifest signs of azotemia.[52] Some also advocate starting continuous renal replacement therapy (CRRT) early, for immunomodulation.

### Modality

The jury is still out on whether CRRT has an edge over intermittent hemodialysis (IHD) in critically ill patients with sepsis. In a recent Cochrane review,[52] no difference in mortality could be demonstrated between the two modalities. However, most studies have excluded patients with significant hypotension and demonstrate that continuous therapies led to improvement in hemodynamic stability and the need for vasopressors prompting them to concede that CRRT may be the preferred mode in very unstable patients. The potential benefits include: better fluid management, temperature control, acid- base-electrolyte control, provision of adequate nutrition, cardiac support, protective lung support, brain protection with preservation of cerebral perfusion and decrease of intracranial pressure, bone marrow protection, blood detoxification and liver support.[51] New concepts and technologies are evolving everyday. One needs to wait and see how the new hybrid technologies like slow low efficiency dialysis (SLED) fare, especially with regards to modulation of the immune system.

### Dosing

Ronco *et al*.[53] show that higher treatment doses in sepsis improve survival. The outcome of patients undergoing continuous veno-venous hemofiltration (CVVH) at doses of 20, 35 and 45 ml / kg / hour were compared. Survival was better in the 35 and 45 ml / kg / hour group as compared to the 20 ml / kg / hour. In the subgroup of patients with sepsis (11-14% patients), there was a trend towards an improved survival even between 35 and 45 ml / kg / hour groups.

The VA/NIH acute renal failure trial network[54] tested this hypothesis in a large number of patients. About 615 patients were treated with CVVHDF at a dose of 20 or 35 ml / kg / hour. They could not show any difference in survival or outcome in the two arms.

### High adsorption hemofiltration

Modern high flux membranes which can remove molecules of 30-40 kD should logically be capable of removing significant amounts of inflammatory mediators. This gives rise to the interesting prospect of cytokine removal by CRRT. However, a clinical study using CVVH at filtration rates of up to 2.6 l / hour could not demonstrate a significant lowering of serum levels of several cytokines, such as interleukin (IL)-1ß, IL-6, IL-8, IL-10, and tumor necrosis factor (TNF). However, within the first hour after placement of a new membrane into the circuit there was a fall in cytokine levels.[55] Ronco utilized this information in a pilot study of 12 patients with sepsis undergoing CVVH, where the AN69 filter was changed every three hours in a nine-hour treatment period. This resulted in a reduction of IL 8 and IL 10 levels and a faster weaning from vasopressor support.[56] However, with the current evidence available, use of CRRT in the absence of AKI cannot yet be justified.

### Hemadsorption

Hemadsorption utilizes adsorbents like charcoal and resins, which can remove solutes by a variety of forces including hydrophobic interactions, electrostatic attraction, hydrogen bonding or van der Waals forces. By altering the structure of solid phase sorbents, selectivity can be achieved. The sorbents have been made more bio-compatible and are attractive adjuncts for cytokine removal in sepsis. A systematic review by Cruz *et al*.[57] finds that polymyxin-B hemoadsorption had beneficial effects on mean arterial pressure, vasopressor use, oxygenation and mortality in sepsis.

### Coupled plasma filtration adsorption (CPFA)

CPFA involves separation of plasma, which is subjected to removal of inflammatory mediators by adsorption over activated charcoal and subsequent hemodialysis. In a pilot trial in 10 patients with septic shock, using a cross-over design, Ronco *et al*,[58] showed a more rapid reduction in vasopressor requirement during 10 hours of CPFA compared to 10 hours of CVVHDF.

### High volume hemofiltration (HVH)

In 2003, Ronco and Bellomo introduced the ‘peak-concentration’ hypothesis, changing the meaning of solute clearance from simple elimination, to immunomodulation by cutting off the heads of the increased and imbalanced levels of pro-inflammatory as well as anti-inflammatory mediators, by hemofiltration.[59]

There have been few randomized trials in septic shock. Cole *et al.* conducted a crossover trial in 11 patients with septic shock and multi-organ failure.[60] Eight hours of HVH at 6 l / hour lead to some reduction of complement levels (C3a and C5a), and more importantly, a rapid decline in vasopressor requirements, as compared to standard CVVH at 1 l / hour. This advantage, however, was not discernible after 24 hours.

To definitely determine the benefit of HVH, the European multicenter high volume in intensive care (IVOIRE) study, targeted to include more than 460 patients with septic shock and AKI, comparing 35 ml / kg / hour with 70 ml / kg / hour, is recruiting patients.[61]

### High cut- off (HCO) hemofiltration

An alternative way of removing mediators would be to use a high cut-off membrane, porous enough to remove larger molecules (about 60 kD). Encouraged by success in *in vitro* experiments and animal studies, a Phase II trial was conducted in 30 patients with septic shock utilizing HCO in CVVH, at an ultrafiltration rate of 2.51 / hour.[62] The study not only showed significant decrease of IL-6 and IL-1 levels but also more clinically important reduction of dosage of norepinephrine. A reduction in simplified acute physiology II (APACHE II) scores were observed in patients treated with HCO-CVVH as compared with conventional CVVH at the same dose. The only setback, as expected, was a significant albumin loss, observed during higher ultrafiltration rates. This can be offset somewhat by using HCO in a diffusive instead of convective process.

### Bio- artificial renal assist device (RAD)

One of the most remarkable recent developments in the field of CRRT has been the development of a renal assist device by Humes *et al.* This entails a cartridge in which 0.5 to 1.0 × 10^8^ non-autologous human renal tubule cells are grown along the inner surface of hollow fibers arranged inside. This device is arranged in a series with the hemofilter in the extracorporeal blood circuit. The ultrafiltrate from the hemofilter passes through the RAD and renal cells therein not only re-absorb and eliminate molecules from the ultrafiltrate, but also perform the metabolic, immunoregulatory and endocrinologic functions normally done by the kidneys.

After many animal experiments and cautious Phase I trials, a multi-center Phase II RCT was conducted in 58 critically ill patients recently. The RAD group had 40 patients of whom 73% had sepsis, and the CRRT only group had 18, of whom 67% had sepsis. On day 28, the mortality rate was 33% in the RAD group versus 61% in the CRRT group and the survival benefit was enhanced at 180 days. The relative risk of death in these patients, with most having at least three organ failures, was 50% less in the RAD group.[63]

## Facilitating renal repair and recovery

Since majority of the patients with sepsis already have some degree of renal damage before the time of detection, by conventional means (RIFLE criteria) it seems logical to give serious consideration to facilitating recovery of renal function.

The modality of RRT may play a role in this regard. The BEST study investigators showed that in an international multi-center trial, patients who were treated with intermittent hemodialysis had a significantly lower likelihood of recovering kidney function, when compared with those treated with CRRT.[64] Moreover, with the use of RAD, renal recovery has been shown to be further accelerated. At the end of 180 days, 3% in the RAD group as compared to 6% in the CRRT group remained dialysis-dependant.[63]

A range of molecules are now under evaluation for their potential regenerative and pro-proliferate effects. These include growth factors: insulin like growth factor, hepatocyte growth factor, and vascular endothelial growth factor; molecules with anti- apoptotic activity: erythropoietin; pro- epithelial and antifibrotic activity: BMP-7 and molecules which can promote renal tubule formation and even accelerate repair: NGAL.[50]

## Conclusion

Sepsis-induced AKI is assuming a distinct identity of its own with a unique pathophysiologic mechanism, behavior and outcome. A better understanding of these will enable us to develop targeted therapeutic strategies. Newer methods, which allow us to detect AKI early, may make these therapies more fruitful. This may encourage us to revisit some of the discarded molecules which may have failed in the past due to late administration. Targeted therapy at the molecular level seems the way forward but is still in its infancy. At present, many of the preventive measures of septic AKI are an offshoot of better global management strategies of the sepsis syndrome. Many modifications of extracorporeal blood purification methods, which are being improvised, seem promising. They presage something beyond simple replacement of renal function, maybe a modulation of the sepsis cascade. These are exciting times and one envisages a lot of progress in the field of septic AKI in the near future.
